# *Leclercia adecarboxylata* as Emerging Pathogen in Human Infections: Clinical Features and Antimicrobial Susceptibility Testing

**DOI:** 10.3390/pathogens10111399

**Published:** 2021-10-28

**Authors:** Souheil Zayet, Stephane Lang, Pauline Garnier, Alix Pierron, Julie Plantin, Lynda Toko, Pierre-Yves Royer, Marc Villemain, Timothée Klopfenstein, Vincent Gendrin

**Affiliations:** 1 Infectious Disease Department, Nord Franche-Comté Hospital, 90400 Trevenans, France; stephane.lang.12@gmail.com (S.L.); alix.pierron@hnfc.fr (A.P.); lynda.toko@hnfc.fr (L.T.); pierre-yves.royer@hnfc.fr (P.-Y.R.); timothee.klopfenstein@hnfc.fr (T.K.); vincent.gendrin@hnfc.fr (V.G.); 2 Microbiology Department, Nord Franche-Comté Hospital, 90400 Trevenans, France; pauline.garnier@hnfc.fr (P.G.); julie.plantin@hnfc.fr (J.P.); marc.villemain@hnfc.fr (M.V.)

**Keywords:** *Leclercia adecarboxylata*, emerging pathogen, MALDI-TOF, clinical features, antimicrobial susceptibility

## Abstract

(1) Background: *Leclercia adecarboxylata* (*L. adecarboxylata*) is a gram-negative bacillus of the Enterobacteriaceae family, which is uncommonly isolated from clinical specimens. *L. adecarboxylata* is considered as an aquatic opportunistic pathogen and most of the human infections are polymicrobial and usually occur in immunocompromised hosts. (2) Methods: In this retrospective study, we included all *L. adecarboxylata* strains since the introduction of MALDI-TOF MS in the Microbiology Department of *Nord Franche-Comté* Hospital, France (from 1 March 2015 to 31 July 2019). We studied demographic characteristics, comorbidities, characteristics of the current infection and outcome as well as antimicrobial susceptibility testing in all isolates. (3) Results: A total of 8 samples were identified (in 6 patients (4M/2F), with a recurrent *L. adecarboxylata* infection in 2 patients). The patients’ mean age was 66.2 years (range: 19–84). All patients were considered as immunocompetent, except a peritoneal dialysis patient with kidney transplantation. An exposition to an aquatic environment was identified in one patient. The most prevalent clinical feature was catheter-associated male urinary tract infection (in 3 cases) followed by ventilator-associated pneumonia (in 2 cases). One of 6 patients presented *L. adecarboxylata* bacteremia. *L. adecarboxylata* was part of a polymicrobial infection in 4 patients. The isolates showed a high susceptibility to all tested antibiotics, except one strain, which was resistant to fosfomycin. All patients with *L. adecarboxylata* infection were treated with antibiotics with a favorable outcome. (4) Conclusion: This study confirms the pathogenicity of *L. adecarboxylata*, even in immunocompetent patients, with a high susceptibility to antibiotics.

## 1. Introduction

*Leclercia adecarboxylata* (*L. adecarboxylata*) was first described by H. Leclerc, in 1962, and previously known as ‘Enteric group 41′ or *Escherichia *adecarboxylata (*E. adecarboxylata*) [1]. Based on nucleic acid and protein electrophoretic techniques, *E. adecarboxylata* was separated from the ‘*Enterobacter agglomerans*’ complex by Izard el al. [2] to which it had been assigned temporarily, and renamed officially as *L. adecarboxylata* by Tamura et al. [3], in 1987. The reclassification was enabled by the emergence of more sensitive testing methods such as DNA hybridization and computer identification studies [4]. *L. adecarboxylata* is a motile, gram-negative bacterium belonging to the Enterobacteriaceae family and sharing it most of its characteristics such as facultative-anaerobic, oxidase-negative, mesophilic, peritrich-flagellated bacilli [2,3]. It is mainly isolated from food, water and environmental sources or animal specimens but has been recognized as an emerging opportunistic pathogen. It can also be isolated from clinical specimens including blood, stool, sputum, urines and wound pus. With new identification methods such as matrix-assisted laser desorption ionization-time of flight mass spectrometry (MALDI-TOF MS), which is preferred for precise species identification over conventional methods, it is currently possible to obtain an accurate identification [5,6,7] of this pathogen. *L. adecarboxylata* is a ‘novel’ rare human pathogen, mostly affecting immunocompromised individuals or causing polymicrobial infections in immunocompetent patients. Although *L. adecarboxylata* is currently susceptible to the common antibiotics, extended-spectrum β-lactamase (ESBL)-producing strains have recently been reported.

We reported, herein, a case series of *L. adecarboxylata* infections among 6 patients (identified in 8 samples), since the advancement of MALDI-TOF MS in our facility.

## 2. Methods

### 2.1. Study Design

We retrospectively studied all *L. adecarboxylata* strains in (Microbiology Department of *Nord Franche-Comté Hospital*), France, from 1 March 2015 to 31 July 2019.

### 2.2. Patient’s Investigations and Baseline Characteristics

We collected demographic characteristics (age, sex), exposition (swimming or not), comorbidities, as well as characteristics of the current infection and outcome from patients’ medical records: episode number and recurrence, clinical feature, CRP value at admission (normal range < 5 mg/L), bacteremia associated or not, antimicrobial drugs used for treatment and evolution).

For each patient, the Charlson Comorbidity Index (CCI) was calculated to provide a score assessing the real weight of comorbidities on the patient’s outcome at the time of diagnosis [8]. 

### 2.3. Identification and Antimicrobial Susceptibility Testing

In the enrolled samples, we noted replicate number identified, mono or polymicrobial infection with associated baterium (bacterial species) and identification scores of bacterial strains. The strains were identified directly after inoculation of samples on the appropriate agar medium, except the dialysis fluid, which was detected negative after incubation. The strain was identified after incubation on *Schaedler broth* + *0.02*% *agar* (+ *Vit*. *K3*) (BioMérieux, Lyon, France) and on aerobic and anaerobic blood culture bottles (Bact/ALERT^®^ FA Plus (aerobic) and FN Plus (anaerobic) bottles, BioMérieux, Lyon, France). Liquid mediums were then inoculated on the standard agar plate. *L. adecarboxylata* was identified using MALDI-TOF MS with an identification score of 2.0 (Table 1). For all samples, direct detection and identification (from agar medium, without extraction) used MALDI-TOF MS (MALDI Biotyper-Microflex^®^, Bruker Daltonics, Bremen, Germany) with Tryptic soy agar with 5% sheep blood, except urine with UriSelect 4 Medium, Bio-Rad, a non-selective chromogenic agar medium.

Antibiotic susceptibility was tested by the disc diffusion assay (disc and Mueller Hinton agar: Biorad, Marnes-la-Coquette, France). CASFM/EUCAST breakpoints were used for the interpretation (Table 2). 

### 2.4. Data Analysis

Continuous variables were expressed as mean and ranges or extremes. Categorical variables were expressed as a number (%). We used the SPSS v24.0 software (IBM, Armonk, NY, USA).

### 2.5. Ethics Statement

This study was sponsored by *Nord Franche-Comté Hospital*, France and designed in accordance with the declaration of Helsinki. Due to the retrospective nature of the study, the Ethics Committee *Nord Franche-Comté Hospital,* France determined that patients’ consent was not required. We have made sure to keep patients’ data confidential and compliance with the Declaration of Helsinki.

## 3. Results

Over a period of 4 years, a total of 8 samples were identified (Table 1). Six patients were diagnosed with *L. adecarboxylata* infection (2 patients presented a recurrent infection with the same presentation). The patients’ mean age was 66.2 years (range: 19–84) with a male predominance (4M/2F). All patients were considered as immunocompetent, except a peritoneal dialysis patient with kidney transplantation. Median CCI was 4.3 (range: 0–8). An exposition to an aquatic environment was identified in one swimmer. All patients presented initially with fever and were hospitalized in conventional medical departments, except one patient who was transferred to intensive care unit (ICU) to be mechanically ventilated for acute respiratory distress syndrome (ARDS). The most prevalent clinical feature was catheter-associated male urinary tract infection (UTI) (in 3 episodes) followed by ventilator-associated pneumonia (in 2 episodes), and in one case, respectively, peritoneal dialysis peritonitis, corneal abscess and vascular prosthetic graft infection.

The median CRP value (in 6 of 8 infection episodes) was 88.9 mg/L (range: 11–272 mg/L). *L. adecarboxylata* was isolated from different samples: 7 of 8 samples in fluids (dialysate, contact lens fluid, bronchial aspiration and in 2 samples and urine in 3 samples) and one isolate in prosthetic vascular graft. Direct examination identified in 6 of 8 patients (75%) gram-negative bacilli. One of 6 patients presented with secondary *L. adecarboxylata* bacteraemia where the source of infection was peritonitis in a patient receiving peritoneal dialysis.

*L. adecarboxylata* was part of a polymicrobial infection in 4 patients. The most common co-infecting organism was *Enterococcus *spp. in 3 patients. *Cutibacterium acnes, Fusarium* spp., *Staphylococcus epidermidis, Klebsilla oxytoca* and *Pseudomonas aeruginosa* were the other co-infecting organisms.

In this case series, all strains of *L. adecarboxylata* were susceptible to penicillins, cephalosporins, carbapenems, aminoglycosides, fluoroquinolones, trimethoprim/sulfamethoxazole (SMX-TMP) and nitrofurantoin. Only one strain was resistant to fosfomycin (Table 2). Two patients presented a recurrent infection with *L. adecarboxylata* (one patient with VAP and one catheter-associated UTI, initially non-treated). All patients presenting one infection episode received antibiotic treatment with favorable outcome. Only one patient with second episode of VAP died due to multiple organ failure.

## 4. Discussion

In this case series of *L. adecarboxylata* infections, we highlighted the pathogenicity of this aquatic agent in human pathology since the advancements in microbiology of high-resolution methods such as MALDI-TOF MS, which led to accurate identification in early diagnosis and distinction of *L. adecarboxylata* from *Escherichia* spp. [5,6,7].

*L. adecarboxylata* is a ubiquitous microorganism, found in aquatic environments but also in soil and in the commensal gut flora of certain animals [9]. In our study, a recent exposition to an aquatic environment was identified in one patient (professional swimmer presenting with corneal abscess with keratitis), such as few cases in medical literature [9]. Until now, this bacterium was considered a low-virulence pathogen with uncertain pathogenicity in human infections [6,9,10]. In our 8 samples, *L. adecarboxylata* was considered as a clinically significant pathogen based on clinical presentations, except for 2 episodes (one patient with VAP and the second with UTI) when clinicians considered it as an asymptomatic carriage or colonization and they have been mistreated. This could probably explain the recurrence of the infection in these two patients and further emphasize the pathogenicity of this agent.

In 2019, Spiegelhauer et al. reported a literature cases review demonstrating its pathogenicity in 74 patients [5]. The majority of these cases had been reported in immunocompromised patients, unlike our patients where the 5 of 6 were immunocompetent; only one patient was considered as immunocompromised following kidney transplantation, although peritoneal dialysis could explain this infection episode given the high prevalence of infections in these patients [7,11,12,13,14].

*L. adecarboxylata* is implicated in cases which involve endocarditis [15,16], catheter-related bacteremia [10,17,18], bacteremia and cellulitis [5,6,9,19,20], urinary tract infections [6,21], pneumonia [5,22] and bacterial peritonitis, especially in peritoneal dialysis patient [7,11,12,13,14], which was the case for the majority of our clinical presentations. *L. adecarboxylata* was most often found as a monomicrobial infection in immunocompromised patients, and as part of a polymicrobial infection in immunocompetent patients [5,12].

In the literature review, the described isolates showed a high susceptibility to antibiotics [5,6]. In our study, this organism showed good susceptibility to common antibiotics and patients were treated successfully with them. In another case series of *L. adecarboxylata* bacteremia, all isolates were susceptible to most of the tested antibiotics and the rare resistances were limited to first-generation cephalosporin and SMX-TMP [20]. To the best of our knowledge, antibiotic-resistant *L. adecarboxylata* strains have only been reported in few cases in human pathology. Of these cases, only three were extended beta-lactamase producer isolates and considered as extended-spectrum beta-lactamase Enterobacteriaceae; one encoded SHV-type beta-lactamases in a 58-year-old man with acute myeloid leukemia [23], the second harbored bla (TEM-1) and bla (CTX-M) group 1 and intl1 genes (dfrA12-orfF-aadA2) as genetic determinants for resistance in a 47-year-old female with breast cancer and catheter-related bacteremia [18] and the third was ESBL-producing a multidrug-resistant *L. adecarboxylata* strain in a 50-year-old female with end-stage renal disease [10].

Recently, Garza-Gonzalez et al. reported an outbreak of carbapenem-resistant *L. adecarboxylata* associated with the unintended use of contaminated total parental nutrition. All the isolates were carbapenemase producers and positive for bla(NDM-1), bla(TEM-1B) and bla(SHV-12) genes [24]. Choudhary et al. also reported the first isolation and characterization of multidrug-resistant Leclercia species from animal clinical case from India [25].

Finally, there are a few limitations of this study: (i) the number of patients is very limited; (ii) the 8 samples have not been tested for all antibiotics at once; in relation to small sample issue, the statistical of the Table 2 can not be established and we can not conclude that 100% isolates were susceptible to all antibiotics.

## 5. Conclusions

To conclude, we think that our data are relevant to clinical and microbiological evidence of *L. adecarboxylata* infection. This study confirms the pathogenicity of *L. adecarboxylata* in human pathology, even in immunocompetent patients. Drug resistance does not seem to be a common issue in *L. adecarboxylata* infections yet. Regarding to the limited number of the reported cases and in our case series, further studies are needed to better elucidate the antibiotic susceptibilities of this emerging agent.

## Figures and Tables

**Table 1:** Patient clinical and microbiological characteristics at baseline in patients with *Leclercia adecarboxylata* infections.

Patient/ Infection Episodes		Age/Sex	CCI/Immunosuppression	Swimming	Clinical Features	CRP (mg/)	Specimens/Replicates (n)	Direct Examination/Culture/Numeration	Polymicobial (Associated Bacterium)/Identification Score	Bacteremia	Pathogenicity/Recurrence	Treatment	Evolution
1		71/F	6/Kidney transplantation	ND	Peritoneal dialysis peritonitis	14	Dialysis fluid [4]	Yes/Yes/ND	No/2.44; 2.42; 2.41; 2.43	Yes	Yes	Amoxicillin	Recovery
2		19/F	0/No	Yes	Corneal abscess with superficial punctate keratitis	ND	Contact lens fluid [1]	Yes/Yes/ND	Yes (*Cutibacterium acnes; Fusarium* spp.; *Klebsiella oxytoca; Candida* spp.)/2.36	No	Yes	Cirofloxacine/Tobramycine	Recovery
3	1st infection	68/M	3/No	ND	Ventilator-associated pneumonia/ARDS	272	Bronchial aspiration [1]	Yes/Yes/10^5^	No (oropharyngeal flora)/2.6	No	No (1st episode) Yes(2nd episode) /Yes	Abstention	Death
	2nd infection				Ventilator-associated pneumonia/ARDS	101	Bronchial aspiration [3]	Yes/Yes/10^7^	Yes *(Enterococcus faecium;Staphylococcus epidermidis) +* oropharyngeal flora/2.52; 2.39; 1.99			Cefotaxime/Linezolid	
4	1st infection	74/M	4/No	ND	Catheter-associated male urinary Tract Infection (BPH)	23	Urine [1]	No/Yes/10^5^	No/2.44	No	No (1st episode) Yes(2nd episode) /Yes	Abstention (urinary catheter removal)	Recovery
	2nd infection				Catheter-associated male urinary Tract Infection (BPH)	11	Urine [1]	Yes/Yes/10^7^				Cefotaxime/then TURP	
5		81/M	8/No	ND	Vascular prosthetic graft infection	112	Iliofemoral., prosthetic vascular graft [2]	Yes/Yes/ND	Yes *(Enterococcus faecium; Pseudomonas aeruginosa)/*2.17; 2.02	No	Yes/Yes	Piperacillin-tazobactam	Recovery
6		84/M	5/No	ND	Catheter-associated male urinary Tract Infection (BPH)	ND	Urine [2]	No/Yes/10^5^	Yes *(Enterococcus faecalis)/*2.47; 2.44	No	Yes/No	Cefotaxime/then TURP	Recovery

Abbreviations (alphabetic order): ARDS: Acute respiratory distress syndrome; BPH: Benign Prostate Hyperplasia; CCI: Charlson comorbidity index; CRP: C–reactive protein; F: Female; M: Male; ND: Not determined; TURP: Transurethral resection of the prostate; UTI: Urinary Tract Infection.

**Table 2:** *Leclercia adecarboxylata* isolates.

	Antibiotics Tested	Susceptible Isolates (%)
Penicillins	Ampicillin	8/8 (100)
	Amoxicillin/clavulanic acid	8/8 (100)
	Ticarcillin/clavulanic acid	5/5 (100)
	Piperacillin/tazobactam	8/8 (100)
Cephalosporins	Cefazolin	3/3 (100)
	Cefoxitin	6/6 (100)
	Ceftriaxone	8/8 (100)
	Cefotaxime	8/8 (100)
	Ceftazidime	8/8 (100)
	Cefepime	8/8 (100)
Carbapenems	Imipenem	8/8 (100)
	Ertapenem	8/8 (100)
Aminoglycosides	Amikacin	8/8 (100)
	Gentamicin	3/3 (100)
	Tobramycin	1/1 (100)
Fluoroquinolones	Nalidix acid	3/3 (100)
	Norfloxacin	5/5 (100)
	Ofloxacin	2/2 (100)
	Ciprofloxacin	6/6 (100)
Folate synthesis inhibitors	Trimethoprim/sulfamethoxazole	3/3 (100)
Nitrofurans	Nitrofurantoin	2/2 (100)
Phosphonic acid	Fosfomycin	1/2 (50)

Not all isolates were tested for all drugs listed. Minimum inhibitory concentration values are not available as disk diffusion testing was employed for susceptibility testing.

## Data Availability

Data available on request due privacy to restrictions. The data presented in this case study are available on request from the corresponding author.
